# Risk factors for insufficient hip distraction for safe central compartment access during hip arthroscopy: retrospective analysis of 677 cases

**DOI:** 10.1093/jhps/hnaf009

**Published:** 2025-02-20

**Authors:** Ching-Chien Chiang, Hao-Che Tang, Cheng-Pang Yang, Huan Sheu, Chieh-An Chuang, Yi-Sheng Chan

**Affiliations:** Department of Orthopedic Surgery, Chang Gung Memorial Hospital, No. 5, Fuxing St., Guishan Dist., Taoyuan 333, Taiwan R.O.C; Department of Orthopedic Surgery, Chang Gung Memorial Hospital, No. 222, Maijin Rd., Anle Dist., Keelung 204, Taiwan R.O.C; Comprehensive Sports Medicine Center, Chang Gung Memorial Hospital, No. 5, Fuxing St., Guishan Dist., Taoyuan 333, Taiwan R.O.C; Department of Orthopedic Surgery, Chang Gung Memorial Hospital, No. 5, Fuxing St., Guishan Dist., Taoyuan 333, Taiwan R.O.C; Comprehensive Sports Medicine Center, Chang Gung Memorial Hospital, No. 5, Fuxing St., Guishan Dist., Taoyuan 333, Taiwan R.O.C; Department of Orthopedic Surgery, Chang Gung Memorial Hospital, No. 5, Fuxing St., Guishan Dist., Taoyuan 333, Taiwan R.O.C; Comprehensive Sports Medicine Center, Chang Gung Memorial Hospital, No. 5, Fuxing St., Guishan Dist., Taoyuan 333, Taiwan R.O.C; Department of Orthopedic Surgery, Chang Gung Memorial Hospital, No. 222, Maijin Rd., Anle Dist., Keelung 204, Taiwan R.O.C; Comprehensive Sports Medicine Center, Chang Gung Memorial Hospital, No. 5, Fuxing St., Guishan Dist., Taoyuan 333, Taiwan R.O.C; Department of Orthopedic Surgery, Chang Gung Memorial Hospital, No. 222, Maijin Rd., Anle Dist., Keelung 204, Taiwan R.O.C; Comprehensive Sports Medicine Center, Chang Gung Memorial Hospital, No. 5, Fuxing St., Guishan Dist., Taoyuan 333, Taiwan R.O.C

## Abstract

Sufficient hip distraction is crucial for the assessment of the central compartment in hip arthroscopic surgery. The aim of this study was to identify the risk factors linked to insufficient hip distraction during hip arthroscopic surgery. We hypothesized that the presence of pincer- or mixed-type femoroacetabular impingement (FAI) could hinder effective hip distraction during the procedure. Inclusion criteria included indication for hip arthroscopy, and persistent symptoms that have not responded to extensive conservative treatment, such as activity adjustments and physical therapy over a period of 6–12 weeks. The enrollment period spanned from January 2003 to May 2021. Data on age, sex, diagnosis of FAI, body mass index, body height, body weight, lateral center-edge angle (LCEA), hip joint space, Tönnis grading, and Beighton score were collected. Among the cases, 34 had insufficient hip distraction while 643 did not. Crude odds ratio analysis revealed that male gender, body height, increased LCEA, the presence of pincer- or mixed-type FAI, and a lower Beighton score were associated with a higher risk of insufficient distraction. Further analyses confirmed that only FAI, sex, and Beighton score remained significant predictors of risk. Adjusted odds ratios indicated a strong association with pincer- or mixed-type FAI. The presence of pincer- or mixed-type FAI is identified as a risk factor for insufficient hip distraction during hip arthroscopic surgery. In high-risk patients, adopting a peripheral compartment approach initially and avoiding hip traction can help prevent traction-related complications.

## Introduction

Over the past decade, hip arthroscopy has emerged as a widely utilized method for addressing both intra- and extra-articular hip pathologies [1, 2]. The hip can be anatomically categorized into the central and peripheral compartments [3]. Sufficient hip distraction is essential for effectively evaluating the central compartment (CC) during hip arthroscopic surgery. However, if the hip joint is resistant to distraction, excessive traction force can lead to traction-related complications [4, 5], including neurovascular injury, direct trauma to neurovascular structures, or compression injury to the perineum.

The CC approach is commonly favored in hip arthroscopy [6]. However, accessing the CC can occasionally be impeded by insufficient distraction, necessitating alternative surgical strategies. These strategies may include exploration of the peripheral or extracapsular compartments or the employment of surgical hip dislocation techniques to address pathologies within the CC.

This research was conducted to delineate the risk factors contributing to insufficient distraction during hip arthroscopy. It was hypothesized that the existence of pincer- or mixed-type femoroacetabular impingement (FAI) could potentially impede effective hip distraction during the procedure.

## Materials and methods

**Figure 1.**
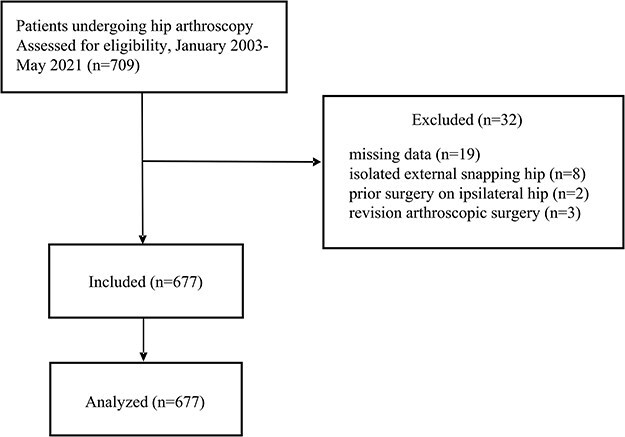
Flow chart diagram of patient recruitment.

We conducted a retrospective analysis of patients who underwent hip arthroscopy under the supervision of the senior author at our institution from January 2003 to May 2021. Patient inclusion criteria included indication for hip arthroscopy, and persistent symptoms that have not responded to extensive conservative treatment, such as activity adjustments and physical therapy over a period of 6–12 weeks. The indications for hip arthroscopy are outlined in Table 1. Exclusion criteria included incomplete medical records, previous surgery undergone on the same hip, or revision arthroscopic surgery. The flowchart diagram depicting patient selection is presented in Fig. 1. Prior to their surgical procedures, all patients were required to provide informed consent, and this process was approved by the Institutional Review Board under reference number 202300893B0.

**Table 1.** Indications of hip arthroscopy and surgical procedures.

Indication of hip arthroscopy	Number of hips (percentage)	Procedure descriptions
FAI	*n* = 470 (66.3%)	Cam or pincer bone hump resection. Acetabular rim trimming. Removal of osteophyte
Labral tear	*n* = 405 (57.1%)	Repair or partial resection of labrum tears
Synovitis	*n* = 52 (7.3%)	Synovectomy
Loose bodies	*n* = 29 (4.1%)	Lavage of central and peripheral compartment
Chondral lesion	*n* = 127 (18.0%)	Cartilage smoothing
Round ligament tear	*n* = 67 (9.4%)	Debridement and synovectomy

### Evaluation of hip lesions

**Figure 2.**
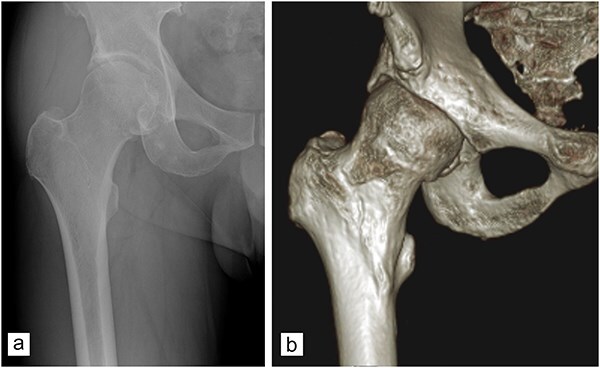
Images from a 68-year-old man with mixed-type FAI. (a) Plain film (AP view) showing a bony structure near the acetabular rim and (b) anterior view of 3D computed tomography reformation showing acetabular overcoverage and bony hump at the femoral head–neck junction.

Demographic information and medical histories of the participants were meticulously recorded. All patients underwent a thorough preoperative evaluation, which comprised a detailed medical history, physical examination, anteroposterior (AP), and frog-leg lateral radiographs of the pelvis, as well as a computerized tomography (CT) with 3D reconstruction of the affected hip joint. Preoperative assessments encompassed the determination of the lateral center-edge angle (LCEA), measurement of the hip joint space width, and evaluation of the Tönnis grade. The LCEA was accurately measured as the angle formed between the vertical axis and the lateral edge of the acetabular bone. The width of the joint space was evaluated on AP radiographs to identify potential joint narrowing, adhering to the methodology outlined by Lequesne *et al*. [7]. The diagnosis of FAI was established based on specific criteria observed in the radiographic assessments. Cam-type FAI was characterized by an *α* angle exceeding 50° or the presence of a pistol-grip deformity [8, 9]. Conversely, the diagnosis of pincer-type FAI was indicated by the observation of acetabular retroversion, coxa profunda, protrusio acetabuli, an LCEA >39°, or an acetabular index equal to or <0° [10, 11]. Furthermore, the 3D CT imaging facilitated a more detailed assessment of the morphological characteristics of the femur and pelvis. All measurements and diagnostic evaluations were performed by a skilled orthopedic surgeon. An illustrative example of the radiological assessment process in a patient, a 68-year-old male diagnosed with mixed-type FAI, is presented in Fig. 2.

### Surgical procedure

**Figure 3.**
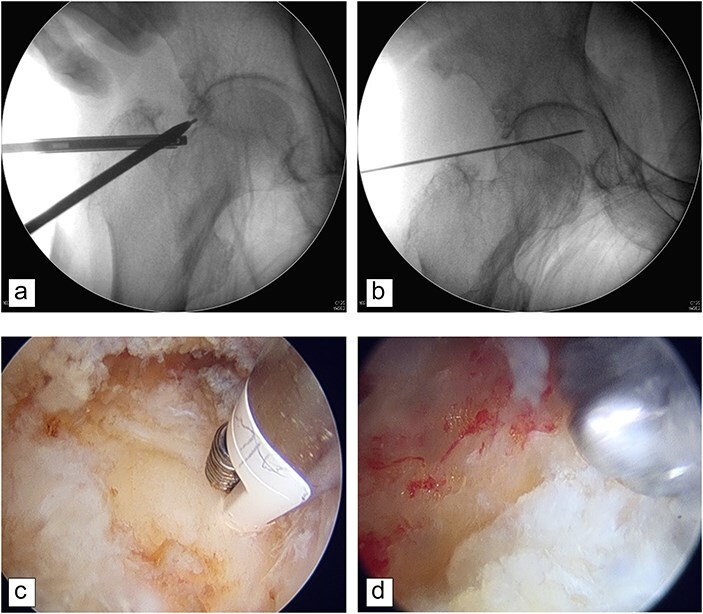
Intraoperative image of the right hip in a traction table. (a) Image intensifier picture of the right hip performed by the peripheral first technique due to insufficient hip distraction. (b) After burring the bony structures, a secondary hip distraction was performed to allow optimal exposure. The needle penetrated the capsule to the CC. (c) Capsulotomy was performed for entry of the peripheral first technique. (d) From the proximal anterolateral portal, acetabular osteoplasty for pincer lesions was performed by the peripheral first technique with the burr.

All procedures involving hip arthroscopy are initiated with a preference for accessing the CC first. Under general anesthesia, patients were positioned supine on a traction table, specifically utilizing the Advanced Supine Hip Positioning System from Smith & Nephew. The positioning of the affected hip was maintained at 0° extension and ∼25° of abduction. Sufficient distraction was critically defined by the capability to attain arthroscopic entry into the hip joint while substantially minimizing the risk of unintended chondrolabral damage. The standard distance for femoroacetabular distraction was established to range from 10 to 15 mm, employing a traction force between 25 and 50 pounds. We initially used hip rotation to negate the sealing effect, thereby enhancing distraction efficacy. If the desired hip distraction could still not be achieved, we then attempted the practice of hip distension using air or fluid during the traction test. This approach successfully facilitated hip distraction in some patients who initially could not achieve adequate distraction. Subsequent to the application of traction, initial access to the CC was facilitated via the anterolateral portal, with the aid of fluoroscopic guidance. In instances where the distraction of the hip did not suffice for secure entry into the CC, a strategy focusing on the peripheral compartment first was adopted, as depicted in Fig. 3. The capsulotomy was performed during the hip arthroscopy.

### Statistical analysis

Demographic and clinical characteristics including sex, age, body height, body weight, body mass index (BMI), radiographic attributes, and Beighton score were evaluated to distinguish between patients achieving sufficient distraction and those with insufficient hip distraction during arthroscopy. Quantitative variables are described using mean values and standard deviations (SDs). The association between categorical variables across the groups was examined using the *χ*^2^ test, whereas comparisons of continuous variables were performed utilizing the two-tailed Student’s *t*-test, with a predefined significance threshold of *P* < .05 for all statistical tests.

Statistical analysis was executed using IBM SPSS Statistics 22 (IBM, Armonk, New York, USA). This included the computation of both crude odds ratio (COR) and adjusted odds ratio (AOR) to assess the risk associated with each variable. The COR for each variable was calculated individually, whereas the AOR was determined through multivariate regression analysis, controlling for potential confounders among the independent variables, and performed backward selection on the model. Results including the odds ratio, 95% confidence intervals, and associated *P*-values are detailed subsequently.

## Results

During the specified study interval, a total of 653 patients underwent hip arthroscopy. Due to incomplete records, 10 patients were excluded, resulting in a final cohort of 643 patients (comprising 301 males and 342 females) and encompassing 677 hip procedures for analysis. The indications for hip arthroscopy identified within this cohort are itemized in Table 1. The proportion of procedures characterized by insufficient hip distraction was 5.2% (34 of 677 cases). Comparative analysis of demographic and radiological parameters between groups with insufficient and sufficient hip distraction was conducted, with findings delineated in Table 2. Notably, a significantly higher proportion of males was observed in the group with insufficient distraction compared to the sufficient distraction group (68.6% vs. 45.8%, *P *= .009). Furthermore, the average body height was significantly greater in the insufficient distraction group (168.9 cm vs. 164.8 cm, *P *= .008), and the mean LCEA was substantially elevated in this group as well (38.6° vs. 28.1°, *P *< .001). The average Beighton score was markedly lower in the insufficient distraction group compared to the sufficient distraction group (0.14 vs. 0.90, *P *= .005), and a higher prevalence of pincer- or mixed-type FAI was significantly associated with insufficient distraction (*P *< .001). However, no significant disparities were found regarding age, body weight, BMI, joint space width, and Tönnis grade between the groups (Table 2).

**Table 2.** Comparison between insufficient or insufficient distraction with demographic and clinical variables.

Variable	With insufficient distraction	Without insufficient distraction	*P*-value
Sex (*n*, %) Male	23 (67.6%)	295 (45.8%)	.011*
Female	11 (32.4%)	348 (54.2%)	
Age (years)			.685
Mean (SD)	43.2 (13.7)	42.2 (13.8)	
Minimum; maximum	17; 67	13; 80	
Body height (cm)			.008*
Mean (SD)	168.9 (10.5)	164.8 (8.8)	
Minimum; maximum	138.7; 192	140.3; 195.1	
Body weight (kg)			.070
Mean (SD)	68.6 (13.3)	64.8 (12.7)	
Minimum; maximum	41; 95.1	40.2; 112	
BMI (kg/m^2^)			.527
Mean (SD)	24.1 (4.7)	23.7 (3.6)	
Minimum; maximum	15.9; 37.3	15.8; 37.0	
LCEA (°)			
Mean (SD)	38.6 (11.6)	28.1 (6.4)	<.001*
Minimum; maximum	25.4; 86.8	18.5; 54.3	
Hip joint space (mm)			.370
Mean (SD)	4.6 (1.0)	4.4 (0.9)	
Minimum; maximum	2.6; 6.6	2.2; 6.6	
Tönnis grade			
0	22 (64.7%)	435 (67.7%)	.792
1	12 (35.3%)	208 (32.3%)	
FAI (*n*, %)	34 (100.0%)	436 (67.8%)	
Cam (*n*, %)	18 (53.0%)	355 (55.2%)	
Pincer (*n*, %)	2 (5.9%)	2 (0.3%)	
Mixed (*n*, %)	14 (41.1%)	79 (12.3%)	
FAI Others	18 (52.9%)	562 (87.4%)	<.001*
Pincer/mixed	16 (47.1%)	81 (12.6%)	
Beighton score			
Mean (SD)	0.14 (0.50)	0.90 (1.58)	.005*
Minimum; maximum	0; 2	0; 7	

* *P *< .05.

The analysis of COR for each variable revealed several associations with an increased risk of insufficient hip distraction, including male gender (COR 2.58, *P *= .111), greater body height (COR 1.053, *P *= .009), increased LCEA (COR 1.151, *P *< .001), the presence of pincer- or mixed-type FAI (COR 6.64, *P *< .001), and a reduced Beighton score (COR 0.48, *P *= 0.023), shown in Table 3. After calculating the COR for the variables, we included all variables in the model and performed backward selection. In the end, only the variables: sex, FAI, and Beighton score remained in the model. Subsequent adjustment of odds ratios (AOR) for confounding variables highlighted a significant correlation of the presence of pincer- or mixed-type FAI (AOR 4.677, *P *< .001) with the heightened risk of insufficient hip distraction. These pivotal findings are concisely encapsulated in Table 4.

**Table 3.** COR for factors associated with insufficient hip distraction.

Variable	*n*	COR (95% CI)	*P*-value
Sex			
Male	318	2.58 (1.24–5.36)	.011*
Female	359	Reference	
Age	677	1.008 (0.983–1.034)	.511
Body height	677	1.053 (1.013–1.095)	.009*
Body weight	677	1.025 (0.999–1.051)	.057
BMI	677	1.036 (0.946–1.135)	.446
LCEA	677	1.151 (1.102–1.203)	<.001*
Hip joint space	677	1.306 (0.869–1.962)	.199
Tonnis grade			
0	457	Reference	
1	220	1.24 (0.63–2.5)	.547
FAI			
Others	580	Reference	
Pincer/mixed	97	6.64 (3.29–13.4)	<.001*
Beighton score	677	0.48 (0.255–0.904)	.023*

* *P *< .05.

**Table 4.** AOR for factors associated with insufficient hip distraction.

Variable	*n*	AOR (95% CI)	*P*-value
Sex			
Male	318	1.803 (0.846–3.842)	.127
Female	359		
FAI			
Others	580		
Pincer/mixed	97	4.677 (2.264–9.663)	<.001*
Beighton score	677	0.557 (0.299–1.037)	.065

* *P *< .05.

## Discussion

The present study revealed that the presence of pincer- or mixed-type FAI was identified as a risk factor for insufficient hip distraction during hip arthroscopy. This finding is supported by previous research; for instance, Henak *et al*. demonstrated that patients with FAI required larger traction forces, while Matsuda *et al*. found that larger traction forces were necessary to distract hips with pincer-type FAI. Sufficient distraction of the hip is crucial for the success of a CC approach in hip arthroscopy. Literature suggests that an 8 mm central or 5 mm lateral hip distraction gap on AP fluoroscopy indicates appropriate distraction [12]. However, based on our experience, the standard femoroacetabular distraction distance typically ranged from 10 to 15 mm, with a traction force of 25 to 50 pounds [5, 13, 14]. Tang *et al*. further demonstrated that a lateral gap increase of more than 2.2 times during an unsterile traction test could indicate successful access to the CC of the hip [15]. In some literature, distension of the hip with fluid or air can break the effect of the suction seal and improve distraction [15, 16]. However, distension of the hip with air or fluid was not performed during the unsterile traction test because rotating the hip can frequently disrupt the sealing effect to improve the distraction [6]. In the present study, all the patients underwent hip arthroscopic surgery for both diagnosis and treatment. No immediate complications were noted during the operation. According to the medical record from January 2003 to May 2021 in Chang Gung Memorial Hospital, a total case number of 677 hips were noted. The most common diagnosis was FAI, and the second common was a labral tear. The diagnostic criteria of FAI are based on arthroscopic visualization. Direct visualization of the hip joint allows the surgeon to assess the presence of any bony abnormalities, such as cam or pincer morphology. In cases of confirmed FAI, the surgeon may proceed to address the impinging structures. This may involve removing excess bone (osteoplasty) to alleviate the impingement, repairing or reconstructing the labrum, and addressing any other identified issues. There were three types of FAI: cam, pincer, and combined impingement [17]. Cam-type FAI involves an abnormality in the shape of the femoral head, typically a nonspherical or aspherical contour and is characterized by an abnormal prominence or bump on the femoral head–neck junction. Pincer-type FAI involves excessive coverage of the femoral head by the acetabulum, often due to an overgrowth of bone on the acetabular rim. Mixed type showed the combination of cam- and pincer-type [18]. FAI will cause groin pain and hip stiffness [19, 20]. The excess bony structures near hip joint could result in hip impingement and limited range of motion [20].

Patients with pincer- or mixed-type FAI tended to have a higher chance of insufficient hip distraction. It may be related to joint laxity that men and patients with FAI have a stiffer hip joint. Henak *et al*. reported that patients with FAI required larger traction forces (10–15 kg) [21]. The study also demonstrated that pincer-type FAI showed the smallest volume, only 15 ml, of joint space under hip distraction. Half of the patients with pincer-type FAI (*n* = 2, total 4) developed difficulties with hip distraction in our study. For example, the conclusion of the previous studies by Matsuda *et al*. showed that larger traction forces were needed to distract hips with pincer-type FAI due to acetabular overcoverage [12, 22]. This explanation is reasonable in terms of hip anatomy in that bony overcoverage of the acetabular ring will bottleneck the movement of the femoral head during hip distraction. This result indicated that pincer-type FAI could cause a much stiffer hip.

A smooth and sufficient hip distraction before access to portals was a crucial step during hip arthroscopic surgery. Lall *et al*. concluded that in previous studies, the upper limit of traction force and traction time should be <50 pounds and 2 h, respectively [23]. Traditionally, the hip arthroscopic procedure is often performed via the CC first after sufficient hip distraction space and shifts to the peripheral compartment for complex cases without sufficient hip distraction. In the current study, 5.2% of cases showed insufficient hip distraction during arthroscopy, and the experienced surgeon then shifted to the peripheral compartment approach to avoid unexpected traction-related complications. To our knowledge, few researchers have evaluated insufficient hip distraction in terms of pathophysiology or biomechanical mechanisms.

In the current study, no significant difference was observed in age, BMI, body weight, hip joint space, or Tönnis grading between groups with and without insufficient hip distraction. While the male sex, increased body height, those with pincer- or mixed-type FAI, and lower Beighton score tended to have a higher chance of insufficient hip distraction in terms of COR, only patients with pincer- or mixed-type FAI remained significant after adjusting for other variables in backward selection. For the difference, this can be explained that other variables (sex, body height, LCEA, and Beighton score) may be associated with FAI. In a previous study, Ellenrieder *et al*. showed that factors related to hip traction force in arthroscopy included sex, height, and weight. From the study, men, taller people, and heavier people tended to have a stiffer hip that needed more hip traction forces for sufficient hip joint space [24]. The results of the current study showed a similar trend, where men and taller people had a higher chance of insufficient hip distraction during arthroscopic surgery without controlling the variables.

The LCEA, hip joint space, and Tönnis grading system were used to assess hip radiographic measurements on plain films. Although a significant excess of insufficient hip distraction risk was observed in association with larger LCEA, we found that decreased hip joint space and increased Tönnis grading score were not associated with increased risk of insufficient hip distraction. The LCEA was shown in measuring acetabular coverage, indicating specific forms of hip pathology, such as hip dysplasia and pincer-type FAI [25]. To date, several reports have revealed that increased LCEA is related to increased hip distraction force [26, 27]. This might be explained by the fact that increased LCEA indicated more acetabular coverage on the femoral head and resulted in insufficient hip distraction.

In the current study, decreased Beighton scores were significantly correlated with insufficient hip distraction in terms of COR. Elevated Beighton scores are commonly observed in patients with significant hypermobility, such as those with collagen disorders like Ehlers–Danlos syndrome [28]. It can be expected that the increased elasticity of collagen in the surrounding tissue of hips would require less distraction force. However, it was close to but did not reach significance in terms of AOR. It could be explained that elevated Beighton scores may be associated with other factors, such as gender. Kwon *et al*. have demonstrated that the female appeared to have a high prevalence of generalized joint hypermobility [29].

There were several limitations in the current study. First, the distraction force and the width of the distraction gap were not measured during hip arthroscopy. Second, a relatively small sample size of insufficient hip distraction (*n* = 34) compared to all cases (*n* = 677) was noted. Third, it was a retrospective study without proper control. Lastly, the lack of severity grading for clinical symptoms or imaging of FAI may limit the precision of the analysis.

## Conclusions

The findings of this study robustly indicate that the presence of pincer- or mixed-type FAI constitutes the independent risk factor for insufficient hip distraction during arthroscopic surgery, even after adjusting for confounding variables. These insights underscore the critical need for surgeons to incorporate these risk factors into their preoperative assessment and counseling strategies. A comprehensive understanding and communication of these risks to patients prior to surgery are imperative to inform decision-making and set realistic expectations. Furthermore, this study highlights the importance of considering the peripheral compartment-first approach as a viable and safer alternative strategy for managing patients predisposed to insufficient hip distraction, thereby minimizing the potential for traction-related complications during hip arthroscopy. The adoption of these evidence-based practices could significantly enhance surgical outcomes and patient safety in hip arthroscopy procedures.

## Data Availability

The data that support this research are available from the Chang Gung Memorial Hospital medical records database. Access to the data is restricted due to patient privacy concerns; therefore, permission and a data-sharing agreement are required for access. Researchers interested in obtaining the data should contact the corresponding author for further information regarding the approval process.
